# Expectations regarding eHealth among women with stress urinary incontinence

**DOI:** 10.1007/s00192-018-3849-2

**Published:** 2018-12-29

**Authors:** Lotte Firet, Doreth Teunissen, Carmen Verhoeks, Antoine Lagro-Janssen

**Affiliations:** Department of Primary and Community Care, Radboud Institute for Health Sciences, Radboud University Medical Center, Nijmegen, the Netherlands

**Keywords:** eHealth, Expectations, Stress urinary incontinence, Women, Pelvic floor muscle training, Qualitative research

## Abstract

**Introduction and hypothesis:**

Stress urinary incontinence (SUI) is a common condition with a major impact on quality of life (QoL). Various factors prevent women from seeking help. However, eHealth (Internet-based therapy) with pelvic floor muscle training (PFMT) is an effective and satisfying intervention for these women. We hypothesize that women with symptoms after regular therapy will profit from eHealth. This study explores the expectations regarding an eHealth intervention among women who still suffer from SUI despite treatment.

**Methods:**

A qualitative study with semistructured interviews was conducted using a grounded theory approach. The study included women with SUI who had ever sought help for their condition.

**Results:**

Thirteen women were interviewed, most whom had experience with PFMT and still suffered from moderate-to-severe incontinence. Two themes emerged from data analysis: the need to meet, and eHealth as a tool to bridge obstacles. Women greatly emphasized that a healthcare professional, preferably one they know, should be available with eHealth. Several women indicated that the absence of personal contact caused lack of trust in success. However, several women were willing to use eHealth because its anonymity and flexibility could overcome obstacles in regular care.

**Conclusions:**

eHealth based on PFMT is currently not a preferable treatment modality for women who still suffer from SUI despite treatment. eHealth cannot act as a substitute for their positive experience with personal contact. Some women are willing to use eHealth because of its advantages over regular care. Future experiences with eHealth might enable women with SUI to trust digital care.

## Introduction

Stress urinary incontinence (SUI) is a common condition in women, which has a significant impact on their lives. The International Continence Society (ICS) defined SUI as the complaint of any involuntary urinary leakage on effort or exertion or sneezing or coughing [1]. SUI prevalence figures vary widely due to methodological differences among studies. The Norwegian Epidemiology of Incontinence in the County of Nord-Trondelag (EPINCONT) study showed a prevalence between 30 and 60% in middle-aged women [2], with a peak among women between 40 and 49 years of age. While SUI is not an alarming condition, it has financial, social, and psychological consequences for women. In the United States, for example, women have to pay out-of-pocket for routine management, such as absorbent products or physiotherapy [3]. They commonly feel ashamed or insecure because of their incontinence, and these feelings could affect their participation in social activities and their quality of life (QoL) [4].

Predominant risk factors for SUI are pregnancy and vaginal delivery, as they impair the pelvic floor muscles [5]. Pelvic floor muscle training (PFMT), therefore, is recommended as effective first-line treatment [6], with the rationale being, in short, that by repeatedly contracting the pelvic floor muscle, the tissue that fails to support the urethra and bladder neck increases in strength, stiffness, and endurance; in addition, women learn to time the contraction because effective contraction is needed before and during effort or exertion [6].

Though effective treatment for SUI is available, only a minority (15–38%) of women appear to be taking advantage of these possibilities [7, 8]. Some women who develop SUI after delivery do not seek help because they consider incontinence to be a normal consequence of giving birth [9, 10]. Other factors that prevent women from seeking help are that they lack knowledge about treatment options, consider urinary incontinence (UI) a consequence of aging, or feel too embarrassed to ask for help [8, 11, 12]. Factors restraining women from seeking help affect the number of women with SUI who eventually receive treatment [11].

Internet-based therapy (eHealth) with PFMT might provide a solution to overcoming barriers that prevent women from seeking help. eHealth consisting of a 3-month Web-based intervention with unsupervised PFMT has shown to be effective in improving treatment of incontinence [13–15]. A main outcome from a qualitative study on women’s experiences with this eHealth intervention showed they felt that their complaints had been acknowledged. Although their previous help-seeking behavior was not described in detail, women reported that several barriers to seeking help had been taken away by eHealth, such as feeling embarrassed to talk about it with their physician [16].

Women’s expectations regarding eHealth in dealing with mental health problems revealed that they thought the anonymity of eHealth would be a major advantage. The absence of personal contact, however, was considered to have a negative impact on women’s motivation to adhere to an eHealth intervention [17]. It is important to study expectations regarding a PFMT-based eHealth intervention, because women’s expectations about PFMT treatment effect are strongly associated with successful treatment [18]. Despite the success of PFMT, it does not reduce or stop incontinence in all women. The exact number of women who, despite treatment, still suffer from SUI is unknown, particularly because longer-term cure rates of PFMT have not been studied extensively [6]. We hypothesized that eHealth might be an effective new treatment modality for these women. Therefore, their expectations regarding eHealth need to be studied to examine whether they would make use of it. Our study aimed to explore expectations regarding eHealth intervention among women who still suffered from SUI despite treatment.

## Materials and methods

Semistructured interviews were carried out among women with SUI to create in-depth knowledge of their expectations regarding eHealth in the light of their previous experiences with treatment. The Consolidated Criteria for Reporting Qualitative Research (COREQ) [19] were applied to this study.

### Participants

Participants were recruited from two general practices in different cities in the eastern part of The Netherlands. Two general practitioners (GPs) purposively selected patients with SUI registered in their electronic medical records, with variability in age, severity of incontinence, education, and duration of symptoms. We defined the following inclusion criteria: female patients, age ≥ 18 years, experiencing predominantly SUI, a history of asking for and receiving help regarding SUI at least once, no history of serious illness, and able to communicate in Dutch. The patients were contacted by their own GP by telephone. The GPs gave a full explanation of the study and asked patients for their approval to pass on their contact details to the researcher (CV). Patients were then contacted by the researcher by telephone to arrange an appointment. Prior to the interviews, all participants were provided with written information, and the interviewer gave a brief explanation about the design of the eHealth intervention. Women were informed that eHealth was defined as therapy via the Internet with home-based PFMT. They were also told there would be no face-to-face contact with a therapist during intervention but that digital communication was possible. All women gave their informed consent.

### Data collection

Semistructured, face-to-face interviews were held, and background data were collected. The validated Severity Index [20] and the Incontinence Impact Questionnaire (IIQ) [21] were used to assess incontinence severity and impact on QoL, respectively. Severity Index scores corresponded with different levels of incontinence severity: 1–2 = slight; 3–5 = moderate; 6–8 = severe. The items of the IIQ short form were divided into the following categories: physical activity (items 1 and 2); travel (items 3 and 4); social/relationships (item 5); emotional health (items 6 and 7). The total score on the IIQ ranged from 0 to 100, with a higher score corresponding to a higher impact on QoL.

Interviews were conducted in July 2016 by a trained medical student (CV) who had no relation with the participants. The interview guide was based on the literature and on the authors’ expertise (DT and ALJ) (Appendix, Table 2). The following topics were addressed: experiences with treatment for SUI; reasons for unsuccessful treatment; expectations regarding an eHealth intervention for SUI; personal support during an eHealth intervention; general preferences for SUI treatment. Depending on the participants’ preference, interviews were held either at their home or at the general practice. Before interviews were held, two pilot interviews were conducted. During the interviews, no field notes were made. Interviews lasted between 20 and 40 min, and no repeat interviews were carried out.

### Data analysis

Interviews were audio-recorded and transcribed verbatim anonymously. Participants were not asked to comment on the transcripts. The grounded-theory approach was used to convert raw data (transcripts) into structured themes that build a theory about expectations women have about eHealth for SUI. The grounded-theory approach is an often-used method to study specific behavior or a specific idea in a population [22]. Two researchers (CV and LS) independently labeled the interview fragments with codes and compared the codes afterward. They used the Atlas.ti (version 7.1.5.) software for this process. After discussion had taken place, the codes were gradually merged into broader categories. A third researcher (LF) read all transcripts to familiarize herself with them, as well as codes and categories. Overarching themes then emerged from the existing categories through intensive discussion with the research committee (all authors). During this process, the researcher (LF) repeatedly compared conceptual themes with transcripts to check whether they would embrace all our data. Finally, agreement was reached on the final themes. We performed no member checking. Though data saturation was reached after 11 interviews, two more interviews were conducted, as we found it ethically objectionable to cancel these scheduled interviews. Important findings in the text are illustrated with quotes from participants, who are identified by identification number, age, and incontinence severity (slight, moderate, or severe); few, several, many, most, or all indicate that 1–3, 4–6, 7–9, 10–12, or 13 participants, respectively, shared an opinion.

### Ethical approval

The Research Ethics Committee of the Radboud University Medical Center, Nijmegen, approved this study (CMO region Arnhem and Nijmegen, The Netherlands, registration number 2016-2625).

## Results

Seventeen eligible patients were approached to participate in the study; four declined because of lack of time. All women had children, and seven were aged >60 years. Most had had experiences with PFMT but still suffered from moderate to severe incontinence (Table 1). Six women had received therapy within the last 2 years. Most women felt capable of handling a computer.

**Table 1:** Participant characteristics

Variables	No. (%)
Age in years	
40–59	6 (46)
60–79	4 (31)
≥80	3 (23)
Education^a^	
Primary	1 (8)
Level 1–4	11 (84)
Level 5–7	1 (8)
Marital status	
Married/living together	8 (62)
Divorced/widowed	5 (38)
Profession	
Employed	4 (31)
Unemployed	5 (38)
Retired	4 (31)
Duration of symptoms (years)	
≤2 years	3 (23)
2–5 years	3 (23)
≥5 years	7 (54)
Severity Index	
Slight	1 (8)
Moderate	6 (46)
Severe	6 (46)
Quality of life (according to IIQ score)^b^	
Good (score ≤ 50)	12 (92)
Poor (≥ 70)	1 (8)
Healthcare professional attended for SUI^c^	
General practitioner	11
Physiotherapist	5
Urogynecologist	3
Experience with PFMT	10 (77)
Weekly computer usage (h)	
≤1	3 (23)
1–10	5 (38)
11–20	5 (38)

*IIQ* Incontinence Impact Questionnaire, *PFMT* pelvic floor muscle training, *SUI* stress urinary incontinence

^a^Level 1–4 = preparatory secondary vocational education, or senior secondary vocational education and training, or senior general secondary education; level 65–7 = universities of applied science or research

^b^Urinary-incontinence-related quality of life according to IIQ score: ≤ 50 = good; 50–70 = average; ≥ 70 = poor

^c^No percentages provided, as some women attended more than one professional

Two themes emerged from data analysis: first, women’s need to meet, which meant that most women preferred to have personal contact in an eHealth intervention and that some, therefore, would not use eHealth. Second, eHealth as a tool to bridge obstacles, meaning that several women were willing to use eHealth because of its perceived advantages that could help bypass obstacles in regular care.

### Need to meet

A predominant view among interviewees was their need for personal contact during therapy for SUI, and the absence of personal contact in eHealth as a stand-alone therapy caused several women to reject it. Women appreciated the contact they had with their GP, physiotherapist, or nurse practitioner when they sought help for the first time. After they had overcome barriers to seeking help, they felt at ease and trusted their healthcare provider because he or she discussed SUI openly with them and normalized the problem.

“I was glad that I went to see my GP; she made me feel at ease and she gave me the feeling that I was not exaggerating things by coming to see her.” (ID2, 51 years, moderate)

During eHealth therapy, most women greatly emphasized the importance of the possibility of contacting a professional if there were any problems. Several women stressed the necessity of this being one and the same person, or at least someone they knew and trusted. The preferred response time from this person during an eHealth intervention was 1 day at most, whereas one woman preferred 24/7 availability, although she knew this would be too costly. Although several women mentioned e-mail, chat, or telephone as possible ways of communication, most women preferred face-to-face contact, because they found communication easier if things could be discussed back and forth. Several women also expressed their resistance to eHealth, because they felt they were computer illiterate, feared being hacked, or resented the digitization of society in general.

“You’d better go to someone and tell them about things; that’s easier than writing down a whole story.’”(ID13, 74 years, severe)

“I don’t feel confident with that thing, the computer, and with all the technology these days. [...] I prefer not to do it [start with eHealth].” (ID1, 68 years, moderate)

Several women thought that a healthcare professional was also needed to provide support during eHealth treatment. Women were concerned that with eHealth as stand-alone therapy, the two following problems could occur: pelvic floor muscle exercises might be performed wrongly if they received no personal feedback; they might lose their motivation for the training program if there was no one to motivate them personally. Several women encountered these problems in regular PFMT, either finding the exercises too difficult or hard to integrate them into their daily lives.

“Pelvic floor muscle exercises are pretty tough. [...] It’s easy to pick the wrong muscles although you might be thinking you’re doing well. It would be nice to have an expert to check it; the computer cannot do that.” (ID 5, 52 years, moderate)

“She [GP] called me after she’d given me the exercises; she encouraged me to do the exercises every day or the muscles would weaken. I have to admit that her message stayed with me.” (ID3, 53 years, moderate)

Next to the need for personal support in eHealth, several women came up with other suggestions to overcome the problems they expected eHealth to raise. Instruction videos showing how to perform the exercises could be embedded; motivation to improve adherence to the training program could be increased by sending automatic reminders or providing tips on how to integrate exercises into daily life. One woman, however, was concerned that digital reminders would unduly emphasize the existence of her incontinence.

“[When undergoing eHealth], I think that you’d be spending too much time thinking about it and focussing on your complaint. That would be no option for me.” (ID12, 82 years, moderate)

### eHealth as a tool to bridge obstacles

Women who were willing to try eHealth emphasized several advantages of the program. They anticipated that eHealth could overcome several obstacles that restrict women from attending a professional to get treatment for SUI. The shame and stigma attached to UI were named as barriers to attending a GP. A few women related that they felt much more ashamed when they had to attend a male GP or consult their GP a second time for the same problem. The idea that a GP would consider SUI to be a minor issue that did not justify a consultation made women reluctant to visit their GP. Anonymity was considered to be an important advantage of eHealth. In particular, those who felt ashamed about SUI commended eHealth because it allows them to perform their exercises in their own homes, or comfort zone, without a healthcare professional’s interference. One woman said that eHealth would enable her to perform the exercises more freely. A few women did not want to have personal support at all during an eHealth training program.

“Yes, it’s a precarious issue, definitely for women, it’s a taboo subject.” (ID6, 70 years, moderate)

“When my complaints become worse, I won’t go [to the GP] again because I find it embarrassing to face that person again; that would make me choose the Internet – it gives you more privacy.” (ID 5, 52 years, moderate)

“I think the advantage [of eHealth] is that you can do it at home, in your own environment. Whereas at the physiotherapist, it’s quite something to lie down or sit on a table and do exercises while someone holds you and checks whether the exercises are going well.” (ID11, 55 years, moderate)

In addition, several women mentioned that eHealth was a solution for socially determined factors that were obstacles to help seeking. These factors included having transportation limits or time restrictions to attend a healthcare professional because women were caregivers. eHealth gave them the opportunity to perform exercises on their own time and at their own pace. This flexibility was also helpful for several women who experienced time limits during PFMT sessions because using eHealth would enable them to reread information. Most women, therefore—including those who would not use eHealth for themselves, expressed the wish that information be provided in a brief and clear manner, supported by visual material. One woman related that eHealth would give her the opportunity to recall the exercises that she had learnt from her physiotherapist, so she could customize her therapy.

“I think that you would allow yourself more time if you do the training program on the Internet than if you have personal contact, because then, you’re mostly more time bound: quick, hurry up. So I suppose that you would be more relaxed.” (ID4, 48 years, moderate)

Women who said they were used to checking the Internet regularly for information on health problems appreciated eHealth for SUI as a source of information. For them, eHealth was a logical step in this digitalized era. Women mentioned the direct access to information about incontinence as an advantage of eHealth for SUI, as it explains that SUI is very common, thus normalizing the problem. A few women suggested that this topic could be addressed in eHealth by videos of other women’s experiences with incontinence or by embedding the possibility for peer support. Making SUI a common problem could also take away women’s concerns about underlying conditions relating to their incontinence, such as prolapse or gynecological cancer. These concerns made a few women reluctant to seek help. A few women mentioned that a physical examination by a GP was needed prior to starting eHealth to alleviate these concerns.

“Everyone is on the Internet, so this [eHealth] fits into these digital times.” (ID12, 82 years, moderate)

“I think I repressed my worries for a very long time. My anxiety was there wouldn’t be anything serious, would there? Maybe cancer or a disease like that [...] This made me postpone seeking help.” (ID11, 55 years, moderate)

Some ideas about treatment options for SUI prevented women from seeking help, and eHealth could provide them with information about therapeutic possibilities. Several women with comorbidity said that their age and condition made them think twice before they sought help because they were resisting interventions, and some of them, therefore, accepted their incontinence. Surgery for SUI was not a treatment option for several women because they had heard about bad experiences from relatives. A few women appreciated that, with eHealth, you have direct access to information about therapy when you forget to ask your GP, for example. Another woman who would not use eHealth herself emphasized its importance for others because if women learned more about incontinence, they would be more likely to be encouraged to seek help.

“Maybe eHealth could provide information about more [treatment] options or possibilities, those things you forget to ask [your GP].” (ID4, 48 years, moderate)

“Women should be informed that if they have problems after delivery, or if they have a prolapse, before it gets worse […] Go to your doctor! Talk about it.”(ID9, 84 years, severe)

## Discussion

This study shows that for women who still suffer from SUI despite treatment, eHealth is not preferred as a stand-alone therapy, because most of them feel they need to meet a healthcare professional in person. This need to meet was based on their positive experiences with face-to-face meetings during previous therapy. The women thought that contact with a professional should be available as a service in eHealth and that support should be provided to enhance a successful outcome. A face-to-face meeting with a professional, however, was not a panacea for all women. As eHealth is anonymous and flexible, several women were willing to use it because it helps lower the threshold they experienced in regular care. eHealth, furthermore, could improve knowledge of incontinence by providing background information and giving explanations about treatment options.

The disadvantages of eHealth as a stand-alone therapy, reflected by the need to meet, were also shown in other studies [16, 17, 23]. In times of need, women like to get in touch with a person they know and trust and with whom they communicate face to face. However, eHealth has the potential to create a satisfying relationship with a therapist who communicates with them remotely [16, 24]. For example, Bjork et al. showed that women who used an eHealth intervention for SUI felt they established a relationship with their urotherapist, even if the urotherapist was available only by e-mail. This relationship was described as personal and distant, in which women felt acknowledged by the therapist [16]. According to Fletcher-Tomenius and Vossler, trust is a multidimensional, complex concept that is essential in any relationship. eHealth could enhance trust because of its anonymity [25], with the absence of face-to-face contact lowering the threshold for discussing a problem and speeding up relation building.

eHealth lowers the threshold to seeking help because of its anonymity and flexibility [16, 17]. The factors that women in this study mentioned as preventing them from seeking help were in line with other studies on such behavior regarding incontinence [11, 12]. For example, shame was named as an obstacle, and the anonymity of eHealth could be useful to overcome this. Previous research shows that women prefer the anonymity of eHealth because they do not have to discuss their problem openly with someone and, therefore, feel less exposed [16, 17].

These advantages of eHealth do not quite match our women’s need for personal contact, which could be explained in various ways. One explanation was already mentioned by the women themselves: they based their preference for personal contact in eHealth on their positive experiences with a healthcare professional during previous therapy. Although unsupervised PFMT is known to be successful [6], a review of qualitative studies on PFMT showed that women find it difficult to achieve bodily knowledge to perform PFMT. When women were assisted by a healthcare professional, they regained their feeling of self-efficacy [26]. The invisibility of the pelvic floor muscle possibly further enhances this feeling of insecurity about practising the method without someone providing feedback.

Another hypothesis is related to the psychological effects of UI. Women with long-term UI feel powerless to stop their urinary leakage and do not feel in control of their bodies [27, 28]. Women in this study made an attempt to stop their incontinence; however, they still suffered at length from UI, and some women eventually accepted their condition. This acceptance could be the result of feeling powerless or of a lack of information about possible treatment options. As researchers recommended in previous studies [27, 28], such women might need the assistance of a healthcare professional who could help them regain their power to control their symptoms.

Although eHealth is not fully embraced by our participants as a new treatment modality, we believe that enthusiasm for eHealth for SUI might increase over time, as eHealth is developing rapidly in many healthcare domains. People with stigmatized illnesses, such as anxiety or depression, use the Internet more frequently to seek information about their disease, and many Internet-delivered health interventions are evolving in the field of psychiatry [29, 30]. We found that computer literacy was related to willingness to use eHealth: women who said they used the Internet regularly to search for health-related information were also in favor of eHealth, whereas those who felt that they were computer illiterate rejected eHealth. The intention to use eHealth is known to increase with experience [31, 32]. In light of the unified theory of acceptance and use of technology, such experience will stimulate the belief that eHealth will help (performance expectancy), increase self-efficacy, and convince users of its comfort (effort expectancy). It is likely that more women will experience some form of eHealth in the future, because women are generally inclined to search the Internet for health-related information, and the uptake among older women is also increasing [32, 33]. Together with women’s need to increase their knowledge of incontinence, this might lead to greater exposure to eHealth [26, 28].

This study showed, however, that an eHealth intervention is currently a bridge too far for women who already have experience with SUI treatment. These women might need more trust to rely on the self-sufficient aspects of eHealth. The GP or the pelvic physiotherapist can enhance such trust by applying motivational interviewing to familiarize patients with eHealth, implementing it as a treatment modality within their daily practice. Depending on individual abilities and preferences, a woman can discuss her preferred treatment with her healthcare provider as an act of shared decision making.

Our study’s strength is that it adds directly to clinical practice by showing the eHealth preferences in a group of patients who have not been studied extensively: women who retain symptoms of incontinence after previous therapy. Another strength is that we included women with a variety of demographic characteristics, such as older women and women with lower levels of education, which is important, because it was predominantly young and highly qualified women who were previously studied with respect to eHealth [13, 14]. However, we did not recruit women with severe levels of QoL impairment due to UI. This could have affected results, because these women might feel even less of an urge to seek help [8, 12] and, therefore, might not even consider eHealth as an intervention they would benefit from. Another limitation is that, being a qualitative study, it is not generalizable to all women with SUI. Quantitative research is needed to study the exact number of women who undergo unsuccessful SUI treatment and to examine their expectations regarding eHealth as a next step to addressing their incontinence condition.

In summary, eHealth based on PFMT is currently not a preferred treatment modality for women who suffer from SUI despite prior treatment. Women in our study believe eHealth cannot be a substitute for their positive experiences with personal contact in regular care. Several women, however, are willing to use eHealth because they expect it to gain advantages over regular care. The uptake of eHealth for SUI might increase in the future if more women start using this new treatment modality. Their experiences may possibly help other women to trust digital care delivered by eHealth.

## Appendix 1

**Table 2:** Interview guide

Topics	Examples of questions
Experiences with treatment	*What were your experiences with attending a therapist for your incontinence?* *What restricted or stimulated you to attend them?* *What were your experiences with treatment for SUI?*
Expectations regarding eHealth for SUI	*What do you think of eHealth? Do you have any experiences with eHealth for other health problems? If applicable, how did you feel about it?* *Considering your incontinence, would you expect disadvantage(s) of eHealth therapy? If so, which one(s)? Would you expect advantage(s) of eHealth therapy? If so, which one(s)?* *Do you think you would use eHealth for SUI? Why or why not?* *How would you prefer to receive instructions for the exercises with eHealth therapy?* *Do you have any tips or suggestions for eHealth intervention?*
Personal support and eHealth for SUI	*Would you prefer personal guidance during eHealth therapy? Why or why not?* *If applicable, how should personal guidance with eHealth be arranged?*
Preferences for treatment	*Would you prefer eHealth or regular therapy for SUI? And why do you prefer it? What would be your goal before undergoing treatment?*

*SUI* stress urinary incontinence

## Appendix 2 Consolidated Criteria for Reporting Qualitative Research (COREQ) guidelines

### Domain 1: Personal characteristics

1. Interviewer/facilitator: *C.V. interviewed all women.*
2. Credentials:

- L.F., M.Sc, currently: Ph.D., general practitioner in training
- D.T., Ph.D, M.D.
- C.V., M.Sc
- A.L-J., Ph.D, Em. Prof, M.D.

3. Occupation: occupation of C.V.: *medical student at the time of study*
4. Gender: *all researchers (= authors) were women*
5. Experience and training: *The interviewer was trained in qualitative interviewing by the supervisors (D.T. and A.L-J.).*
6. Relationship established: *There was no relationship established prior to study commencement*.
7. Participant knowledge of the interviewer: What did participants know about the researcher*? The interviewer told participants about the goal of the study and that she was part of a project about eHealth for women with stress urinary incontinence.*
8. Interviewer characteristics: What characteristics were reported about the interviewer/facilitator? *See 2, 4, and 7.*

### Domain 2: Study design

#### Theoretical framework

9. Methodological orientation and theory: *We applied the grounded theory approach to the transcripts, which were constructed after the semistructured interviews had been conducted. The transcripts were coded; these codes were merged into categories, and final themes were constructed from these categories.*

#### Participant selection

10. Sampling: *We selected participants through purposive sampling.*
11. Method of approach: *Participants were recruited from two general practices in different cities in the eastern part of The Netherlands. Two GPs selected patients who were registered with stress urinary incontinence in their electronic medical record. The GPs contacted the patients and provided them with information about the study. Furthermore, they asked permission for contact details to be passed on to the researcher. The researcher contacted the patients by telephone and arranged an appointment.*
12. Sample size: *Thirteen participants*
13. Nonparticipation: *Four women declined because of lack of time.*

#### Setting

14. Setting of data collection: *Depending on the participant’s preference, interviews were held either at their home or at the general practice.*
15. Presence of nonparticipants: *There was no other person present.*
16. Description of sample: *See* Table 1*. All women had children, and seven women were aged >60 years. Most had had experiences with PFMT, but still had a moderate-to-severe degree of incontinence.*

#### Data collection

17. Interview guide: *The interview guide was based on literature and on the supervising committee’s expertise (See Appendix* Table 2*). We performed semistructured interviews. Before the interviews were carried out, the interview guide was pilot tested twice.*
18. Repeat interviews: *Repeat interviews were not carried out.*
19. Audio/visual recording: *We made use of an audio-recorder during the interviews.*
20. Field notes: *No field notes were made.*
21. Duration: *Interviews lasted 25 min, on average.*
22. Data saturation: *Saturation was achieved after 11 interviews. The last two interviews were conducted because appointments for interviews were already set. These interviews revealed no new finding. Data saturation has been discussed.*
23. Transcripts returned: *Transcripts were not returned to participants.*

### Domain 3: Analysis and findings

#### Data analysis

24. Number of data coders. How many data coders coded the data? *Two researchers independently coded the transcripts.*
25. Description of the coding tree: *We did not provide a coding tree. The code list is available from the corresponding author on reasonable request.*
26. Derivation of themes: *Themes were derived from data.*
27. Software: *Atlas.ti version 7.1.5 was used.*
28. Participant checking: *Member checking was not performed.*

#### Reporting

29. Quotations presented: *Yes, quotations are displayed with participant identification number, age, and incontinence severity indicated as slight, moderate, or severe.*
30. Data and findings consistent: *There was consistency between data presented and findings.*
31. Clarity of major themes: *We believe major themes are clearly presented.*
32. Clarity of minor themes: *If there were inconsistencies within themes, we provided nuances within the major themes. For example: “The shame and stigma attached to UI were named as barriers to attending a GP. A few women related that they felt much more ashamed when they had to attend a male GP ...””*

## Abbreviations

GP: General practitioner
IIQ: Incontinence Impact Questionnaire
PFMT: Pelvic floor muscle training
SUI: Stress urinary incontinence
